# Parotidectomy and neck dissection in locally advanced and relapsed cutaneous squamous cell carcinoma of the head and neck region^☆^^☆☆^^★^

**DOI:** 10.1016/j.bjorl.2021.11.007

**Published:** 2021-12-10

**Authors:** Giulianno Molina de Melo, Luiz Henrique Guilherme, Marcel das Neves Palumbo, Marcello Rosano, Murilo Catafesta das Neves, Fabiano Mesquita Callegari, Marcio Abrahao, Onivaldo Cervantes

**Affiliations:** a Universidade Federal de São Paulo, Escola Paulista de Medicina (UNIFESP/EPM), Departamento de Otorrinolaringologia e Cirurgia de Cabeça e Pescoço, São Paulo, SP, Brazil; b Hospital Beneficência Portuguesa de São Paulo, Departamento de Cirurgia de Cabeça e Pescoço, São Paulo, SP, Brazil; c Universidade Federal de São Paulo, Escola Paulista de Medicina (UNIFESP/EPM), Departamento de Patologia, São Paulo, SP, Brazil

**Keywords:** Skin neoplasms, Prognosis, Parotid neoplasms, Survival analysis, Salivary gland diseases

## Abstract

**Objective:**

To investigate the prognostic factors to developing parotid and neck metastasis in locally advanced and relapsed Cutaneous Squamous Cell Carcinoma (CSCC) of the head and neck region.

**Methods:**

Single-center retrospective cohort study enrolling consecutive patients with advanced CSCC from 2009 to 2019. Seventy-four cases were identified. Study variables demographic data, clinical skin tumor stage, neck stage, parotid stage (P stage), surgical treatment features, and parotid, regional, and distant metastases. Survival measures: Overall Survival (OS) and Disease-Specific Survival (DSS).

**Results:**

The study group included 72.9% men (median age, 67 years); 67.5% showed T2/T3 tumors, 90.5% comorbidities, 20.2% immunosuppressed, with median follow-up: 35.8 months. The most frequent skin primary were auricular and eyelid regions, 75% underwent primary resection with flap reconstruction. Parotid metastasis was present in 50%, 32.4% showing parotid extracapsular spread, multivariate analysis found OR = 37.6 of positive parotid metastasis evolving into positive neck metastasis, *p* = 0.001. Occult neck metastasis, neck metastasis, and neck extracapsular spread were observed in 13.5%, 51.3%, and 37.8%, respectively. Kaplan–Meier survival: Clinical T4 versus T1, *p* = 0.028, P1 stage: 30% and 5% survival at 5 and 10 years, P3 stage: 0%, *p* = 0.016; OS and DSS showed negative survival for the parotid metastasis group, *p* = 0.0283.

**Conclusion:**

Our outcomes support a surgically aggressive approach for locally advanced and relapsed CSCC, with partial parotidectomy for P0, total parotidectomy for P1–3, selective I–III neck dissection for all patients and adjuvant radiochemotherapy to appropriately treat these patients with advanced CSCC of the head and neck region.

**Level of evidence:**

II b – Retrospective Cohort Study – Oxford Centre for Evidence-Based Medicine (OCEBM).

## Highlights

• Parotidectomy and neck dissection in locally advanced (laCSCC) and relapsed Cutaneous Squamous Cell Carcinoma (reCSCC) were evaluated.
• Worst survivals were observed in T4, positive P stage and positive parotid metastasis.
• The parotid metastasis was present in 50% with OR = 37.6 to evolve into positive neck metastasis.
• The occult, neck metastasis and neck extracapsular spread rate was 13.5%, 51.3% and 37.8%.
• We propose partial for P0 or total parotidectomy for P1-3 and neck dissection to all these patients.

## Introduction

Cutaneous Squamous Cell Carcinoma (CSCC) is the second-most common neoplasia in humans after basal cell carcinoma; approximately 1.2 million new cases of head and neck skin cancer are estimated to occur in 2040, with 680,000 deaths.^1^ In Brazil, 173,930 new cases of nonmelanoma skin cancer were estimated to occur in 2020, with approximately 2000 deaths.^2^ Approximately 80% of CSCCs are located in the head and neck region, and advanced CSCC has a mortality rate of 20%, although this value may have been underestimated due to the lack of precise data in developing countries in South and Central America.

Advanced CSCC is defined as locally invasive into deep anatomic structures with or without the presence of regional or distant metastasis; described as Locally Advanced (laCSCC) or Metastatic (mCSCC); classified as T3/T4, stage III or IV, where no parotid and local treatment standards are well established; Relapsed CSCC (reCSCC) is defined as multiple recurrences after successful margin-free resection, occurring in the same location as laCSCC.^3^ The estimated incidence is 8000 cases of nodal metastasis and 3000 deaths annually for both laCSCC and mCSCC, with little evidence data to reCSCC, in which is an area with unmet medical needs.^4^

The recommendations for treatment are based on a literature review and guidelines. Generally, primary surgical excision with safety margins and appropriate lymph node chain dissection are necessary for proven positive regional metastasis or in high-risk patients but debate still exists about the clinically negative neck and extension of the parotidectomy.^5^

Parotid metastasis from CSCC is not common, occurring in 1%–5% of all cases of CSCC in the head and neck region, and regional metastasis can occur up to five years after resection of the primary tumor.^6^, ^7^, ^8^ The high-risk clinical features for parotid and neck metastasis from CSCC include size (T); depth; scalp or ear pavilion involvement; immunosuppression; recurrence; and poor differentiation.^9^, ^10^, ^11^, ^12^ Recently, the immunosuppression induced by drug delivery to organ transplant patients, those with autoimmune diseases and other clinical comorbidities (diabetes *mellitus*), has been recognized as an important risk factor for the development of CSCC, with a growing incidence and mortality worldwide.^13^, ^14^

Parotid metastasis from CSCC has an unfavorable prognosis, with lower survival and a higher incidence of locoregional recurrence and occult neck metastasis; however, there is little information about the biological tumoral spread from the parotid to neck nodes.^8^, ^11^, ^15^, ^16^, ^17^ There are some parotid and neck staging systems, denominating it as positive (P+) or negative (P0), or the N1S3 classification, which does not have a consensus worldwide. At present, the most accepted system is the TNM system, although the pN staging evaluation is directly affected by the surgical technique, dissection amount, and the quality of the pathologic examination.^18^, ^19^, ^20^, ^21^, ^22^

To date, no consensus exists regarding parotid treatment and the indication for or required extent of neck dissection in reCSCC and laCSCC; the identification of the risk factors for parotid metastasis in reCSCC and laCSCC can allow for selection of the proper treatment approach for the parotid gland and neck dissection.

## Objective

The objective of this study was to investigate the prognostic factors for developing parotid and neck metastasis in reCSCC and laCSCC of the head and neck region and to evaluate the parotid treatment and neck dissection treatment results.

## Methods

This retrospective study with a cohort case-series design was conducted at a single center, public teaching hospital, with the same surgeons and included referred consecutive patients with laCSCC and reCSCC in the head and neck region with or without parotid or neck metastasis from 2009 to 2019. Institutional ethical review board approval was obtained before beginning this project, and the patients provided informed consent. The Ethics Committee approval number for this study was 0905/2015, CAAE: 48857315.6.0000.5505, in March 2016.

This study was conducted in accordance with the Preferred Reporting of Case Series in Surgery (PROCESS) criteria following the PROCESS guidelines,^23^ the Guidelines for Cohort Studies in Surgery by Agha et al.^24^ and the Standards for Quality Improvement Reporting Excellence Guidelines (SQUIRE 2.0).^25^ The study was conducted in accordance with the Declaration of Helsinki and has been registered under WHO Universal Trial Number (UTN) U1111-1249-0072 and Brazilian Clinical Trials Registry (ReBeC) number RBR-36vxx7.

Anatomopathological evaluation of tissue samples was performed according to university standards. All samples of the primary skin tumors and surgical specimens were re-examined by two experienced pathologists and reclassified according to the TNM 8ed. and WHO definition criteria.^11^, ^20^, ^26^, ^27^

After confirmation of the CSCC diagnosis, descriptions of patients and tissue samples were recorded, including sex, age, comorbidities, date of diagnosis, mean duration of symptoms, surgical procedure, surgical complications, parotid status and neck lymph node status based on clinical and radiological findings, clinical (cTNM) and pathological (pTNM) stages as defined by the TNM 8ed staging system,^20^ margin status (positive, negative),^28^ Perineural Invasion (PNI), Angiolymphatic Invasion (ALI), radiotherapy, chemotherapy data, recurrence data, patient condition and date of last consultation. Although it is not widely used, the parotid staging adopted by our service was performed according to the P stages by O’Brien et al.:^29^ P1, metastatic SCC < 3 cm; P2, tumor 3–6 cm or multiple metastatic parotid nodes; P3: tumor > 6 cm, VII nerve palsy or skull base invasion.

The inclusion criteria were as follows: cutaneous squamous cell carcinoma in the head and neck region, recurrence status and/or advanced primary stage (T3 and T4), T1 and T2 cases that were relapsed were included, referred to our multidisciplinary team in the head and neck cancer department, CSCC histologic variants, with or without parotid or neck metastasis, subjected to surgical treatment with curative intent for the skin primary tumor and/or the tumors (or not) in the parotid and/or in the neck, with a minimum follow-up of six months.

The exclusion criteria were as follows: Merkell cell carcinoma, melanoma and basal cell carcinoma patients, patients lost to follow-up, incomplete clinical data, surgery performed at other institutions on their primary tumor or neck, distant metastasis at first presentation, refusal to participate in the study, refusal to undergo surgical treatment, and surgery not performed due to poor clinical status.

Statistical analyses were conducted with the odds ratios to calculate the risk of exposed cases, a two-proportion test to compare the proportions of two variables, the Chi-Squared test and Student’s *t*-test with a significance of *p* < 0.05 for comparing frequencies, logistic regression analyses with a significance of *p* < 0.05, univariate and multivariate analysis, and Kaplan–Meier survival curve analyses.

## Results

Between 2009 and 2019, we identified a total of 91 patients with advanced cutaneous carcinoma. Eleven patients with basal cell carcinomas were excluded, 3 were lost to follow-up, and 3 others refused surgical treatment. The final group of 74 patients, all with laCSCC or reCSCC in the head and neck region, was designated as the study group and divided according to parotid metastasis status into a positive Parotid Metastasis group (PM) with 37 patients who were compared to the Without Parotid Metastasis (WPM) group with 37 patients.

### Patients and clinical characteristics

The demographic, clinical, and surgical characteristics of the two groups are shown in Table 1.

**Table 1:** Clinical and demographics characteristics of the locally advanced and relapsed cutaneous squamous cell carcinoma of the head and neck region.

Characteristics	Variable	With parotid metastasis – PM (%)	Without parotid metastasis – WPM (%)
Sex	Male	30 (81.1)	24 (64.9)
	Female	7 (18.9)	13 (35.1)
Age (years)	Mean	67.3	66.6
	Median	71	65
Comorbidites	Yes	31 (83.8)	36 (97.3)
	No	6 (16.2)	1 (2.7)
Primary skin location	Auricular	12 (32.4)	5 (13.5)
	Scalp	7 (18.9)	4 (10.8)
	Frontal	4 (10.8)	4 (10.8)
	Around lip	2 (5.4)	2 (5.4)
	Eyelid	7 (18.9)	10 (27.0)
	Malar	4 (10.8)	6 (16.2.)
	Nose	1 (2.7)	6 (16.2)
Clinical T primary	RT1 (<2 cm)	2 (5.4)	9 (24.3)
	RT2 (≥2 cm < 4 cm)	3 (8.1)	23 (62.1)
	T3 (≥4 cm)	21 (56.7)	3 (8.1)
	T4	11 (29.7)	2 (5.4)
Type surgery primary	No	4 (11.1)	0 (0)
	Total resection with flap reconstruction	20 (55.6)	16 (43.2)
	Maxilectomy	0 (0)	4 (10.8)
	Temporalectomy	6 (16.7)	4 (10.8)
	Exenteration	5 (13.9)	5 (13.5)
	Rinectomy	0 (0)	4 (10.8)
	Other	1 (2.8)	4 (10.8)
Type parotid surgery	No parotid surgery	1 (2.7)	22 (59.5)
	Partial	16 (43.2)	11 (29.7)
	Total	20 (54.1)	4 (10.8)
Type neck dissection	No	1 (2.8)	22 (59.5)
	Selective	17 (45.9)	7 (18.9)
	Modified radical	14 (37.8)	7 (18.9)
	Radical	5 (13.5)	1 (2.7)
Pathological stage	I	1 (2.7)	1 (2.7)
	II	3 (8.1)	18 (48.6)
	III	15 (40.5)	13 (35.1)
	IV	18 (48.6)	5 (13.5)
Adjuvant treatment	Yes	33 (89.2)	21 (56.8)
	No	4 (10.8)	16 (43.2)
Adjuvant treatment type	Irradiation alone	25 (67.6)	21 (56.8)
	Chemotherapy alone	0 (0)	0 (0)
	Chemoradiotherapy	8 (21.6)	6 (16.2)
Recurrence	Yes	8 (21.6)	9 (24.3)
	No	29 (78.4)	28 (75.7)
Distant metastasis	Yes	3 (8.1)	5 (13.5)
	No	34 (91.9)	32 (86.4)
Follow-up period (months)	Mean	35.8	30.1
	Median	19	14

RT1 stage, Relapsed T1 stage; RT2 stage, Relapsed T2 stage.

The study group included 72.9% men with a median age of 67 years (range, 23–103 years). The distribution of primary clinical skin T stages, included T1 and T2 relapsed cases, is shown in Table 1. T2 and T3 were more prevalent. The locations of the lesions were auricular, 22.9%; scalp, 14.8%; frontal, 10.8%; eyelid, 22.9%; malar, 13.5%; and nose, 9.4%.

Comorbidities were observed in 90.5% of the patients; 20.2% were immunosuppressed due to some kind of transplant. The mean follow-up was 35.8 months.

### Treatment

The primary advanced skin cancer surgery was total resection with flap reconstruction 48.6%; exenteration 13.5%; temporalectomy 13.5%; and rhinectomy 5.4% (Table 1). Histological differentiation was classified as follows: well, 35.1%; moderate, 43.2%; and poor, 21.6%. The primary skin cancer surgical margins were compromised in 40.5% (Table 3).

The overall incidence of neck metastasis was 32.4%: 51.4% in the PM group and 13.5% in the WPM group (occult). Of these, 22.9% showed positive neck extracapsular spread. The type of neck dissection was selective (45.9%), modified radical (37.8%), or classic radical (8.1%) (Table 1, Table 2).

**Table 2:** Tumor and pathological characteristics of the locally advanced and relapsed cutaneous squamous cell carcinoma of the head and neck region, based on presence or absence of parotid metastasis.

Pathological characteristics	Variable	With parotid metastasis – PM (%)	Without parotid metastasis – WPM (%)
Cutaneous primary	Auricular	12 (32.4)	5 (13.5)
	Scalp	7 (18.9)	4 (10.8)
	Frontal	4 (10.8)	4 (10.8)
	Around lip	2 (5.4)	2 (5.4)
	Eyelid	7 (18.9)	10 (27.0)
	Malar	4 (10.8)	6 (16.2)
	Nose	1 (2.7)	6 (16.2)
Histological differentiation degree	Well	17 (45.0)	9 (24.3)
	Moderately	11 (29.7)	21 (56.8)
	Poor	9 (24.3)	7 (18.9)
Parotid metastasis	Yes	36 (97.3)	1 (2.7)
	No	1 (2.7))	36 (97.3)
Size of parotid metastasis (cm)	0	1 (2.7)	36 (97.3)
	<1	2 (5.4)	0 (0)
	1–3	12 (32.4)	0 (0)
	>3	22 (59.5)	1 (2.7)
Neck metastasis	Yes	19 (51.4)	5 (13.5)
	No	18 (48.6)	32 (86.5)
P stage (cm)	P1 (1–3)	20 (54.1)	0 (0)
	P2 (3–6)	16 (43.2)	1 (2.7)
	P3 (larger than 6)	1 (2.7)	0 (0)
N stage	N0	13 (35.2)	32 (86.5)
	N1	12 (32.4)	2 (5.4)
	N2	12 (32.4)	3 (8.1)
	N3	0 (0)	0 (0)
Lymph node extracapsular spread	Yes	14 (37.8)	3 (8.1)
	No	23 (62.2)	34 (91.9)
Perineural invasion	Yes	14 (37.8)	30 (81.1)
	No	23 (62.2)	7 (18.9)
Angiolymphatic invasion	Yes	12 (32.4)	7 (18.9)
	No	25 (67.6)	30 (81.1)
Parotid metastasis extracapsular spread	Yes	23 (62.1)	1 (2.7)
	No	14 (37.8)	36 (91.9)
Margins	Free	17 (45.9)	26 (70.1)
	Exiguous	4 (10.8)	5 (13.5)
	Compromised	16 (43.3)	6 (16.2)
Pathological stage	I	1 (2.7)	1 (2.7)
	II	3 (8.1)	18 (48.6)
	III	15 (40.5)	13 (35.1)
	IV	18 (48.6)	5 (13.5)

P stage, Parotid stage; N stage, Neck stage.

The overall incidence of parotid metastasis was 50.0% and partial parotidectomy was performed as follows: PM 43.2% and WPM 29.7%, total parotidectomy: PM 54.1% and WPM 10.8%. The parotid P stage was P1 (54.1%), P2 (43.2%), and P3 (2.7%) in the PM group and P1 0%, P2 (2.7% – occult), and P3 (0%) in the WPM group (Table 2). Overall parotid metastasis extracapsular spread was present in 32.4%, and the PM group showed: (a) Total parotidectomy: 54.1%, positive ECE 35.1%, compromised margins: 29.7%; and (b) Partial parotidectomy: 43.2%, positive ECE 27.0%, compromised margins: 27%.

The N stage was N0 (60.8%), N1 (18.9%), and N2 (20.2%). Of these, 22.9% of the patients presented with lymph node extracapsular spread.

Adjuvant treatment was administered to 72.9% of patients; 62.1% underwent external radiotherapy alone, none received brachytherapy, and 18.9% received chemoradiotherapy (Table 1).

### Outcomes

The overall recurrence rate (local and regional) was 22.9%, and distant metastasis was 10.8% (Table 1). In the PM group, 51.4% of the patients also had neck metastasis. The rate of neck occult nodal disease was 13.5% (5/37). The mortality rate related to the disease was 32.4%; the morbidity rate (alive with neoplasia) was 27.0%; and only 40.5% of the patients were alive without disease; thus, the survival rate was 67.6%.

Table 3 shows the univariate analysis, demonstrating differences in relation to extracapsular spread from the neck, extracapsular spread from the parotid, and compromised margins. In Table 4, logistic regression multivariate analysis showed that parotid metastasis extracapsular spread was significantly associated with the development of neck metastasis (OR = 37.61).

**Table 3:** Cox proportional hazards univariate analysis between groups of presence and absence of parotid metastasis of the locally advanced and relapsed cutaneous squamous cell carcinoma of the head and neck region.

		PM		WPM		Total		*p*-Value
		n	%	n	%	n	%	
Comorbidities	No	16	43.2%	11	29.7%	27	36.5%	0.227
	Yes	21	56.8%	26	70.3%	47	63.5%	
Lymph node extracapsular spread	No	23	62.2%	34	91.9%	57	77.0%	0.002
	Yes	14	37.8%	3	8.1%	17	23.0%	
Parotid extracapsular spread	No	14	38.9%	36	97.3%	50	68.5%	<0.001
	Yes	23	62.1%	1	2.7%	23	31.5%	
Angiolinfatics invasion	No	25	67.6%	30	81.1%	55	74.3%	0.183
	Yes	12	32.4%	7	18.9%	19	25.7%	
Perineural invasion	No	23	62.2%	30	81.1%	53	71.6%	0.071
	Yes	14	37.8%	7	18.9%	21	28.4%	
Compromised margins	No	17	45.9%	26	70.3%	43	58.1%	0.034
	Yes	20	54.1%	11	29.7%	31	41.9%	
Exiguous margins	No	33	89.2%	32	86.5%	65	87.8%	0.722
	Yes	4	10.8%	5	13.5%	9	12.2%	
Degree of differentiation	Well	17	45.9%	9	24.3%	26	35.1%	0.107
	Moderately	12	32.4%	20	54.1%	32	43.2%	
	Poor	8	21.6%	8	21.6%	16	21.6%	
Primary tumor margins	Compromised	14	37.8%	15	41.7%	29	39.7%	0.933
	Exiguous	5	13.5%	5	13.9%	10	13.7%	
	Free	18	48.6%	16	44.4%	34	46.6%

**Table 4:** Cox proportional hazards multivariate analysis of the locally advanced and relapsed cutaneous squamous cell carcinoma of the head and neck region to development of neck metastasis.

	Coef. (B)	*p*-Value	Odds ratio		
			OR	Inferior limit	Superior limit
Constant	−1.230	0.003			
Lymph node extracapsular spread	0.422	0,667	1.53	0.22	10.43
Parotid metastasis extracapsular spread	3.627	0.001	37.61	4.30	329.40
Compromised margins	0.140	0.838	1.15	0.30	4.37

The survival curves showed no differences in the comparisons based on the type of parotid surgery, histological differentiation degree, recurrence of primary CSCC in a different location, or pathological final stage (I/II × III/IV) in both groups.

### Disease survival

**Figure 1:**
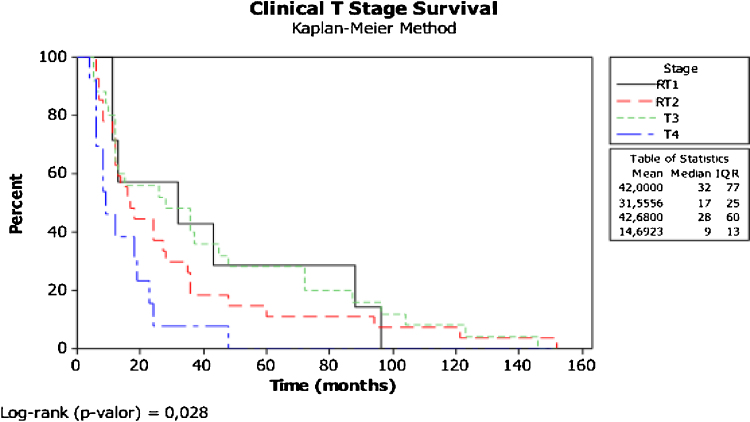
Kaplan–Meier survival curve between clinical T stage (8ed.).

The specific survival curve for clinical T stage (Fig. 1) showed significant differences, worsening as the T stage advanced (worst in T4 vs. T1 *p* = 0.028).

**Figure 2:**
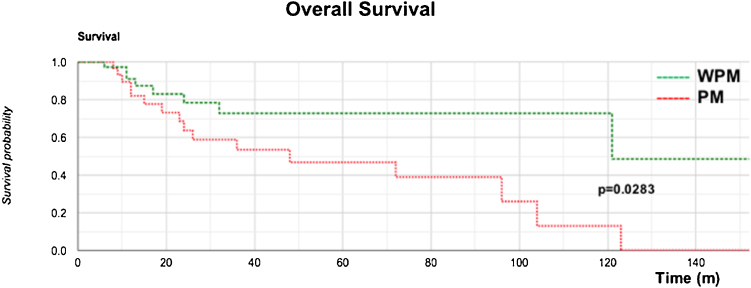
Comparison of Kaplan–Meier overall survival between Groups PM and WPM.

The Kaplan–Meier OS curve showed a significantly worse prognosis (log-rank; *p* = 0.0283) in the PM group (Fig. 2). The OS rates at 3 years, 5 years, and 10 years in the PM group were 58%, 46%, and 13%, respectively, while those in the WPM group were 78%, 72%, and 72%, respectively.

**Figure 3:**
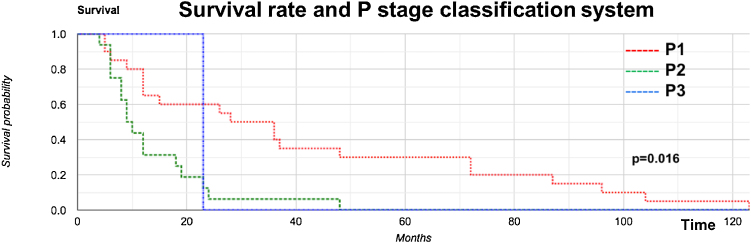
Correlation between survival rate and P stage classification system.

Kaplan–Meier analysis of DSS on the P stage in the PM group showed significant differences: P1 and P2 at 3, 5, and 10 years were 40%, 30%, and 5%, respectively; and 6%, 0%, and 0%, respectively. The values for P3 were 0% in all corresponding periods of time (*p* = 0.016; Fig. 3).

## Discussion

### Synopsis of the new findings

Our cohort study assessed 74 patients with laCSCC and reCSCC of the head and neck region, with a median follow-up of 35.8 months; 50% showed parotid and/or neck metastasis, much higher than the 20%–39% range reported in the literature, explained by health system failures and the continental size of a country located in tropical areas that contributes to advanced clinical stage presentation.^18^, ^30^, ^31^ The majority of the patients were men, with a median age of 67y, with a history of prolonged sun occupational exposure.

In our cases, the prevalent location of the primary CSCC was the auricular and eyelid region, almost 67.5% were relapsed T2 and T3, and some relapsed T1 CSCC evolved with parotid metastasis, probably due to immunosuppression conditions, contrary to some authors (Table 1).^30^, ^32^ In univariate and multivariate analyses, primary tumor site, histopathological characteristics and margins did not affect OS, which was attributed to retrospective data bias.^33^, ^35^ Interestingly, our study found that 90.5% of patients with comorbidities (20% immunosuppressed due to transplanted organs) developed progressive metastases to the parotid/neck.^36^, ^37^, ^38^

In 75% of patients, the main surgery was total primary resection with reconstruction followed by exenteration and temporalectomies, showing an aggressive advanced presentation, similar to other developing countries.^35^ Our data did not find an association between the type of primary surgery and survival, unusual since such a correlation is expected; however, there are some comparable studies.^34^, ^38^, ^39^

In our cohort of patients with no clinically or radiologically evident parotid metastases, we did not use Sentinel Lymph Node Biopsy (SLNB), as there is no institutional protocol approved for this approach in our service, although several papers in the literature currently recommend this less aggressive approach for T2b and T3 tumors; nevertheless, more randomized controlled studies are necessary to validate the SLNB approach, as the actual available data do not indicate it provides any benefit regarding further metastasis or tumor-specific survival.^40^, ^41^, ^42^, ^43^

The surgical margins were compromised in 56.7% in the PM group and only 16.4% in the WPM group (*p* = 0.034), as expected in advanced cases where there was no clear tumor-free tissue intraoperatively, but it did not directly affect survival. The majority of PM group patients had clinical T3/T4 (86.4%) tumors (most laCSCC), unlike the WPM group (most reCSCC), which showed relapsed T1/T2 (86.4%); this biological behavior difference impacted the survival (T4 × T1) curves, with a clearly negative impact on clinical T stage survival (*p* = 0.028) (Fig. 1).

Parotid metastasis from CSCC has an unfavorable prognosis.^17^, ^18^, ^21^, ^44^ Our study reported a 50% incidence of parotid metastasis in laCSCC and reCSCC; 32.4% showed parotid extracapsular spread, with 56.7% having compromised parotid surgical margins, creating a challenge in achieving adequate surgical treatment once the parotid is severely compromised;^10^, ^14^, ^21^, ^36^^,^^45^, ^46^, ^47^, ^48^ otherwise, our isolated occult parotid metastasis rate was only 2.7%, meaning that it is probably safe to observe clinically and radiologically negative parotid gland lymph node cases (Table 2, Table 3). Although no difference was observed among the types of parotid surgery, our overall recurrence rate was 22.9%, most frequent in parotid surgical beds (21.6%).

Our overall rate of occult neck metastasis was 13.5%; in the PM group, 51.3% presented clinical neck metastasis, and in 37.8% of these, extracapsular spread, demonstrating the path of the tumor behavior; once it has established a parotid gland metastasis, it rapidly invades the lymph nodes in the neck. In fact, 64.8% of the PM group had N1–N2 neck compared to 13.5% N1–N2 neck in the WPM group (Table 2); notably, the risk of a positive parotid metastasis evolving into positive neck metastasis was 37.6 times, with *p* = 0.001 (Table 4).

Our OS curve (Fig. 2) demonstrated poor survival for the PM group, with the worst predictors (*p* = 0.0283). Once the parotid showed clinically positive metastasis (P1–3 stages), its disease-specific survival was progressively and severely impacted (Fig. 3) (*p* = 0.016), findings similar to others.^18^, ^31^, ^32^, ^36^^,^^44^, ^45^, ^47^, ^48^, ^49^, ^50^, ^51^

Although 72.9% of our patients underwent adjuvant chemoradiotherapy, this treatment did not positively impact survival, possibly due to the poor response of CSCC to chemoradiation (Table 4),^10^, ^32^, ^35^, ^36^^,^^48^ but chemoradiotherapy is still the standard of care adjuvant treatment in multiple guidelines.^12^, ^21^, ^31^, ^32^^,^^36^, ^52^

Table 5 shows a review of the literature over 20 years regarding overall survival and parotid and neck metastasis in CSCC, including our data.

**Table 5:** Comparison of review literature results in 20 years of similar studies: parotid and neck metastasis, radiotherapy and disease specific survival rates in metastatic SCC of skin to parotid gland.

Authors	Study (year)	Patients (n)	Parotid metastasis (%)	Neck metastasis (%)	Adjuvant Rtx (%)	Survival at 5 years (%)
O’Brien CJ et al.	2001	123	59.3	26	Not mentioned	58
O’Brien CJ et al.	2002	87	75.8	42.1	86	63
Teymoortash A et al.	2002	19	31.5	31	100	52
Chua MS et al.	2002	52	31	Not mentioned	100	65
O’Brien CJ et al.	2002	87	All included	24	86	63
Bron LP et al.	2003	232	43.5	23.7	85.1	75
Jol JA et al.	2003	41	24	39	100	46
Palme CE et al.	2003	126	64.2	35.7	89	68
Dona E et al.	2003	74	50	25	100	72
Veness MJ et al.	2003	167	Not mentioned	All included	87	73
Audet N et al.	2004	56	All included	100	66.1	53
Moore BA et al.	2005	193	42	20.7	100	95
Veness MJ et al.	2005	167	59	41	87	73
Ch’ng S et al.	2006	67	55.2	35.8	67	54
Andruchow JL et al.	2006	322	80.7	19.2	82.9	69
Hinerman RW et al.	2008	117	81.2	18	72	47
Ch'ng S et al.	2008	170	46	29	77	69
Goh RYH et al.	2012	67	61	39	100	72
Ebrahimi A et al.	2012	168	100	19.6	80	89
Košec A et al.	2013	103	24.2	23.3	90	55.7
Makki FM et al.	2013	54	All included	30	87	81
Thom JJ et al.	2014	42	26	31	100	93
Shao A et al.	2014	160	93.7	28.1	80	48
Chen MM et al.	2015	2014	All included	41.3	48.6	71
Bachar G et al.	2016	71	11.3	36.6	100	63.3
Hirshoren N et al.	2017	149	61	79	84	50
Czerwonka L et al.	2017	136	All included	70	79	79
Coombs AC et al.	2018	63	All included	Not mentioned	81	53
Bobin C et al.	2018	35	All included	46.9	85.7	59
Creighton F et al.	2018	62	93.5	37.9	73.8	56
Varra V et al.	2018	76	All included	All included	100	60
Coombs AC et al.	2018	63	All included	Not mentioned	81	78
Present study	2020	74	50	32.4	82.4	67.6
Hasmat S et al.	2020	535	43.9	56.0	65.6	55.1

### Clinical applicability

The high levels of locoregional recurrence and the poor outcomes have guided us to adopt a more aggressive initial approach in these higher-risk patients with laCSCC and reCSCC: (a) To observe the parotid and neck in the some P0 cases; or (b) Partial parotidectomy in high risk P0 cases; and (c) Total parotidectomy in the P1–3 patients. Since the survival is poor in the P1–P3 patients and there is no difference in recurrence and survival between the types of parotidectomy, we chose to reduce the risk of facial nerve complications in the P0 stages.

We also suggest performing (d) Selective (I–III) neck dissection in all P1–3 laCSCC and reCSCC patients to achieve prolonged survival, since it is difficult and hazardous to adopt the “wait and see” protocol in the neck in these high-risk cohorts.

## Conclusion

Our study demonstrated that in laCSCC and reCSCC, 50% of patients had parotid metastasis, 32.4% had extracapsular parotid metastasis spread, 13.5% had overall neck occult metastasis, 51.3% had clinical neck metastasis, and 37.8% had neck extracapsular spread. The risk of positive parotid metastasis evolving into positive neck metastasis was 37.6. A clinical skin T4 tumor and the presence of parotid metastasis negatively impacted patient survival; the P1 stage had 30% and 5% survival at 5 years and 10 years, respectively, and the P3 stage had 0% survival. Once the parotid shows clinically positive metastasis (P1–3 stages), disease-specific survival is progressively and severely impacted.

## Authors’ contributions

Giulianno Molina de Melo: Study concept, study design, data acquisition, data analysis and interpretation, manuscript preparation, manuscript editing and review, final approval of the version to be published and accountable for all aspects of the work.

Luiz Henrique Guilherme: Data acquisition, manuscript editing, final approval of the version to be published and accountable for all aspects of the work.

Marcel das Neves Palumbo: Data acquisition, manuscript preparation, final approval of the version to be published and accountable for all aspects of the work.

Murilo Catafesta das Neves: Data acquisition, manuscript preparation, final approval of the version to be published and accountable for all aspects of the work.

Marcello Rosano: Data acquisition, manuscript preparation, final approval of the version to be published and accountable for all aspects of the work.

Fabiano Mesquita Callegari: Study design, data analysis and interpretation, manuscript preparation, manuscript editing and review, final approval of the version to be published and accountable for all aspects of the work.

Marcio Abrahão: Study design, data analysis and interpretation, manuscript preparation, manuscript editing and review, final approval of the version to be published and accountable for all aspects of the work.

Onivaldo Cervantes: Study design, data analysis and interpretation, manuscript preparation, manuscript editing and review, final approval of the version to be published and accountable for all aspects of the work.

## Funding

The Federal University of Sao Paulo Ethics Committee approval number to this study was 0905/2015, CAAE: 48857315.6.0000.5505, in March 2016.

## Conflicts of interest

The authors declare no conflicts of interest.
